# Influence of the preparation method on the photocatalytic activity of Nd-modified TiO_2_

**DOI:** 10.3762/bjnano.9.43

**Published:** 2018-02-06

**Authors:** Patrycja Parnicka, Paweł Mazierski, Tomasz Grzyb, Wojciech Lisowski, Ewa Kowalska, Bunsho Ohtani, Adriana Zaleska-Medynska, Joanna Nadolna

**Affiliations:** 1 Department of Environmental Technology, University of Gdansk, 80-308 Gdansk, Poland; 2 Department of Rare Earths, Faculty of Chemistry, Adam Mickiewicz University in Poznan, 60-780 Poznan, Poland; 3 Institute of Physical Chemistry, Polish Academy of Sciences,01-224 Warsaw, Poland; 4 Institute for Catalysis, Hokkaido University, Sapporo 001-0021, Japan

**Keywords:** heterogeneous photocatalysis, hydrothermal method, modified TiO_2_, neodymium, sol–hydrothermal method

## Abstract

Nd-modified TiO_2_ photocatalysts have been obtained via hydrothermal (HT) and sol–hydrothermal (SHT) methods. The as-prepared samples were characterized by X-ray diffraction (XRD), BET surface area measurements, scanning electron microscopy (SEM), diffuse reflectance spectroscopy (DRS), luminescence spectroscopy and X-ray photoelectron spectroscopy (XPS). The photocatalytic activity of the synthesized samples was evaluated by the degradation of phenol in aqueous solution under irradiation with UV–vis (λ > 350 nm) and vis (λ > 420 nm) light, as well as by the degradation of gaseous toluene under irradiation with vis (λ_max_ = 415 nm) light. It was found that Nd-modified TiO_2_ is an efficient photocatalyst for the degradation of phenol and toluene under visible light. XPS analysis revealed that the photocatalyst prepared via HT method contains a three-times higher amount of hydroxy groups at the surface layer and a two-times higher amount of surface defects than that obtained by the SHT method. The photocatalytic efficiency of phenol and toluene degradation under vis irradiation in the presence of 0.25% Nd-TiO_2_(HT) reached 0.62 and 3.36 μmol·dm^−1^·min^−1^, respectively. Photocatalytic activity tests in the presence of Nd-TiO_2_ and scavenger confirm that superoxide radicals were responsible for the visible light-induced degradation of the model pollutant in aqueous solution.

## Introduction

Heterogeneous photocatalysis based on titanium dioxide (TiO_2_) has become the focus of numerous studies due to its possible applications in the treatment of air, water and wastewater [1–5] after the photocatalytic splitting of water on TiO_2_ electrodes was discovered by Fujishima and Honda in 1972 [6]. However, serious disadvantages of TiO_2_ are its wide band gap, which requires the use of ultraviolet light irradiation for excitation (the band gap of anatase is about 3.2 eV) and low quantum yield (due to the fast recombination of electron–hole pairs) [7]. These restrictions on its application could be overcome by modifying TiO_2_, which results in increased activity and ability to work under visible light irradiation [8–10].

In recent years, the modification with rare earth (RE) metals has proven to be an efficient method to improve the photocatalytic properties of TiO_2_ and to broaden its absorption to the solar spectrum [8,11–12]. Various Lewis bases can form complexes with RE-modified TiO_2_, which leads to enhanced surface adsorption properties of TiO_2_ and indirectly increases the photocatalytic activity of the photocatalysts [13–14]. Moreover, it has been reported that the presence of RE ions slowed down the rate of the charge-carrier recombination processes [15]. Besides, RE ions, due to the 4f electron structure, can act as conversion luminescent media [14,16–18]. Neodymium (Nd^3+^) ions are well known as one of the more interesting lanthanides due to unique electronic and optical properties [19–20]. Several reports suggested that the transformation of light from near-infrared and visible spectral ranges into ultraviolet wavelengths can be responsible for the photocatalytic activity of RE-modified TiO_2_ [21–22]. In our previous paper, based on action spectra analysis we revealed that Nd-modified TiO_2_ could be excited under visible light in the range of 400 to 480 nm. Nevertheless, it has been proven that the up-conversion process was not responsible for the degradation of phenol under vis irradiation [23]. A similar conclusion based on action spectra analysis was also formulated by us for other RE-TiO_2_ systems activated by visible light [11,14,24].

The photocatalytic activity is strictly affected by physical properties such as crystalline structure and size, specific surface area, and the density of surface hydroxy groups [7]. The impact of the synthesis of TiO_2_ nanoparticles (NPs) on properties and morphology is important for understanding, creating and improving materials for various applications. According to the literature, RE-modified TiO_2_ could be prepared through a wide spectrum of methods, such as sol–gel [18,25], hydrothermal [23,26–27], solvothermal [28], electrospinning [29], co-precipitation [30] and electrochemical [8] methods.

In our previous papers, we presented experimental studies concerning the effect of synthesis methods on lanthanide-modified TiO_2_. In these studies we compared hydrothermal and sol–gel method. The synthesis method significantly affects the structure, optical, luminescence and photocatalytic properties. It was found that all samples (TiO_2_ modified with Y^3+^, Pr^3+^, Er^3+^ and Eu^3+^) obtained through the hydrothermal method approach exhibited a higher photocatalytic activity, while the samples prepared via the sol–gel method approach yielded more luminescence when irradiated with 980 nm photons [24].

Meanwhile, the hydrothermal treatments in combination with the sol–gel method (sol–hydrothermal method) provide an alternative approach for preparing TiO_2_. During the preparation, before the hydrothermal process, a sol is obtained, which may prevent the agglomeration of the nanocrystals [26]. However, information regarding the photocatalytic activity of Nd-modified TiO_2_ NPs preparated by the sol–hydrothermal method is still lacking in the literature.

In view of this, in the present study, we proposed to combine the effects of synthesis route and neodymium modification to improve the photocatalytic activity of TiO_2_ under visible light. The Nd-modified TiO_2_ photocatalysts have been prepared using two different preparation routes, namely hydrothermal and sol–hydrothermal methods. The surface properties of these photocatalysts have been correlated with the preparation method as well as with the photoactivity in two model reactions, degradation of phenol in aqueous solution and degradation of gaseous toluene. Moreover, the photocatalytic mechanism of the Nd-modified photocatalysts was explored using a scavenger test.

## Results and Discussion

Table 1 contains the description of all prepared photocatalysts, including preparation method, content of Nd, BET surface area and crystallite sizes. The amount of Nd precursor (0.25 mol %) was selected based on previous research [10,24].

**Table 1:** Sample label and physicochemical characterization of pristine and Nd- modified TiO_2_ photocatalysts.

sample label	preparation method	content of Nd (mol %)	*S*_BET_ (m^2^/g)	crystallite size (nm)

pristine-TiO_2_(SHT)	sol–hydrothermal	0	106	16.3
0.25% Nd-TiO_2_(SHT)	sol–hydrothermal	0.25	137	14.3
pristine-TiO_2_(HT)	hydrothermal	0	117	8.9
0.25% Nd-TiO_2_(HT)	hydrothermal	0.25	126	7.4

### Structural properties

**Figure 1:**
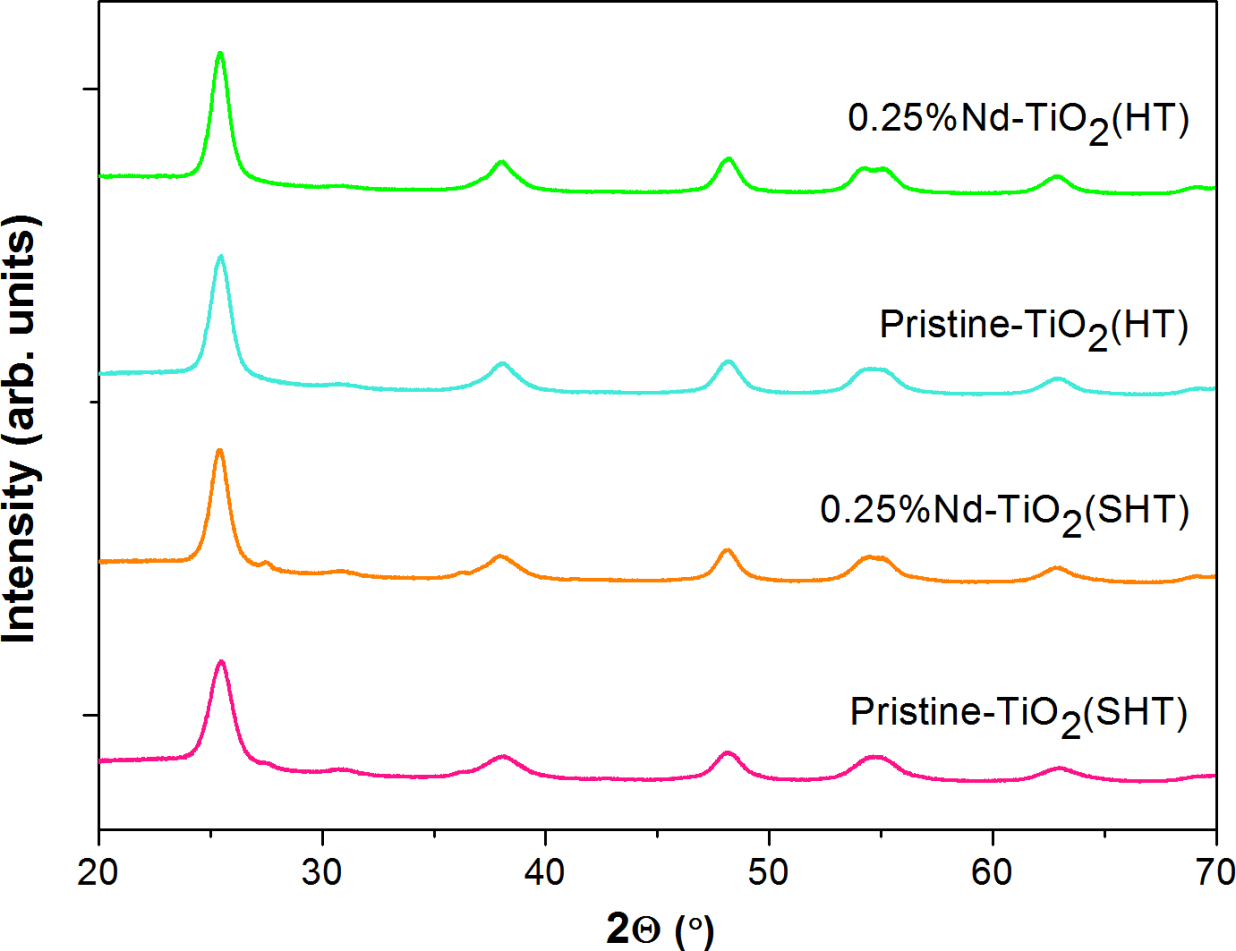
X-ray diffraction patterns of pristine TiO_2_ and Nd-modified TiO_2_ prepared by sol–hydrothermal and hydrothermal methods.

The X-ray diffraction (XRD) technique was used to determined the crystalline phase of pristine TiO_2_ and Nd-modified TiO_2_ NPs. The XRD patterns of the examined samples are shown in Figure 1. In all synthesized photocatalysts, the diffraction pattern presents a group of lines at 2θ values of 25.4, 37.9, 48.1, 54.1, 55.1 and 62.9°, which are characteristic of anatase phase. The phase transformation to rutile has not occurred despite the heat treatment at 450 °C. The absence of peaks corresponding to Nd_2_O_3_ in the XRD patterns can be ascribed to the following phenomena: The content of Nd^3+^ ions was too small or highly dispersed and was below the detection limit of the diffractometer, Nd^3+^ was present in the form of an amorphous phase or Nd^3+^ ions adsorbed either on the titania surface or Nd^3+^ cations were placed inside the titania lattice (titania doping) [24,31].

Using the Scherrer equation, the average crystallite size of anatase was determined and presented in Table 1. For modified samples, 0.25% Nd-TiO_2_(SHT) and 0.25% Nd-TiO_2_(HT) the crystallite sizes were 14.3 and 7.4 nm, respectively. While, for pristine samples, pristine-TiO_2_(SHT) and pristine-TiO_2_(HT) the crystallite sizes were equal to 16.3 and 8.9 nm, respectively. This indicates that the neodymium ions can inhibit the growth of crystallite size of TiO_2_. The photocatalysts prepared by the hydrothermal method showed a higher contraction of crystallites than those obtained by the sol–hydrothermal method. The present study reveals that loading with neodymium ions hindered the increase in grain size of TiO_2_ during hydrothermal synthesis. Our findings correlate with some other reports. A similar average particle size of anatase (compared to the HT samples) was observed by Thomas and co-workers [31]. In their study, NPs of Nd-TiO_2_ were prepared by using titanium(IV) isopropoxide and NdCl_3_ as the titanium and neodymium source in the hydrothermal reaction at 180 °C for 2 h. The surface area of all as-prepared samples ranged from 106 to 137 m^2^/g (Table 1). All Nd-modified photocatalysts showed higher BET surface area than the pristine samples. The highest BET surface area was observed for the 0.25% Nd-TiO_2_(SHT) sample. According to literature data and our previous investigation, the presence of lanthanides in the obtained samples contributed to a decrease in size of the crystallites, which probably caused an increase in the specific surface area of photocatalysts [24,32–33].

The difference in ion radii (0.99 Å for Nd^3+^ and 0.75 Å for Ti^4+^) suggests that Nd^3+^ ions cannot be effectively incorporated into the TiO_2_ lattice. Additionally, a full inclusion of Nd^3+^ ions may be hindered by the low coordination number of Ti^4+^ ions (CN = 6), because Nd^3+^ ions prefer sites with CN = 8 or 9. Therefore, it is reasonable to assume that Nd-containing species are localized at the surface of TiO_2_ [34]. Xie et al. [35] have reported that during the hydrothermal reaction, Nd^3+^ and O^2−^ ions could form a Nd–O oxide on the superficial layer of the TiO_2_ particle by a chemical bonding process and Nd^3+^ ions mainly occur in the form of Ti–O–Nd in the Nd-TiO_2_ compound. The formation of Ti–O–Nd bonds probably restricts direct contact between TiO_2_ crystallites and stabilizes the anatase phase and crystallite growth [25,35].

### Morphology

**Figure 2:**
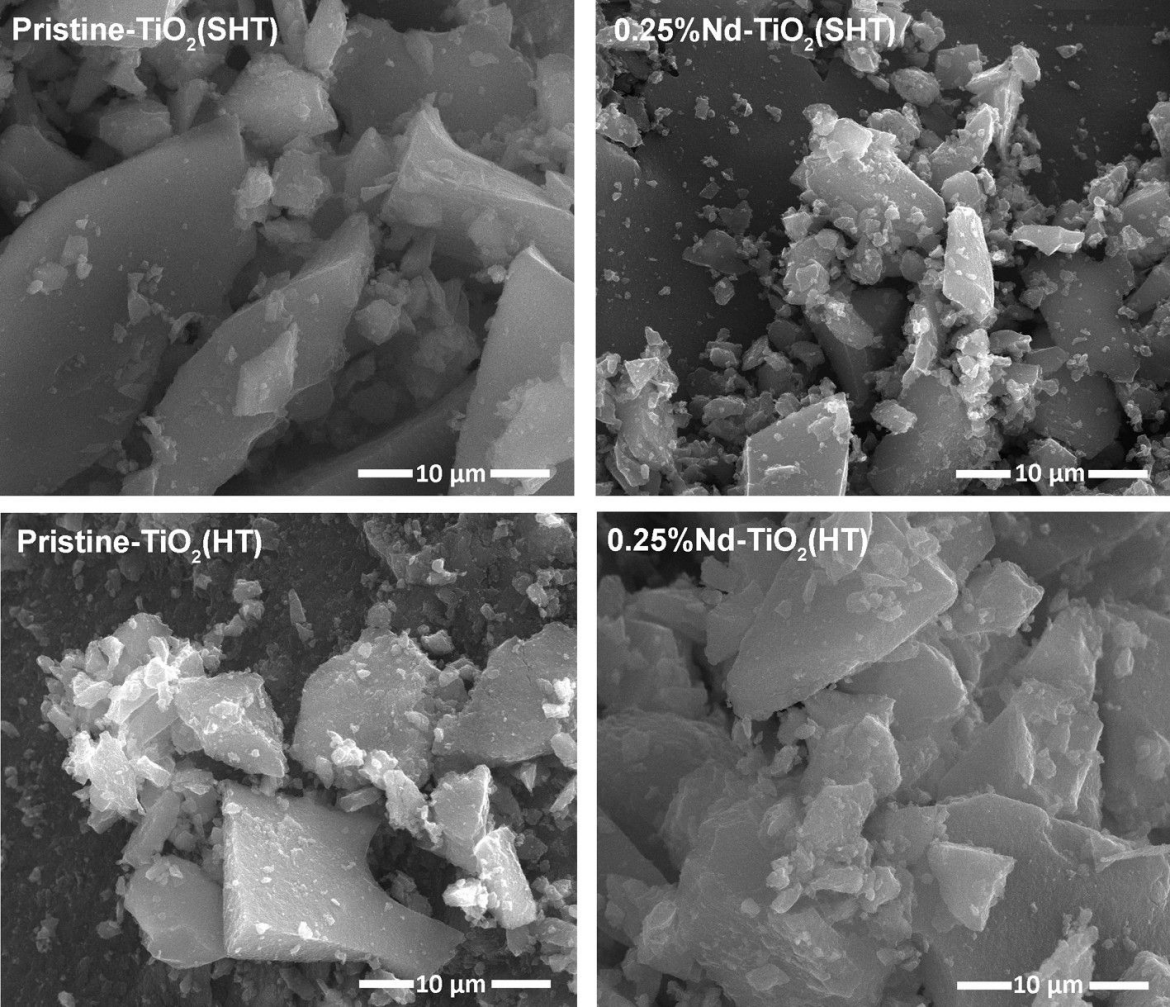
SEM images of TiO_2_ and Nd-modified TiO_2_.

Surface morphology of the obtained photocatalysts was studied using scanning electron microscopy (SEM). Figure 2 shows a SEM image of pristine TiO_2_ and Nd-modified TiO_2_, and an irregular shape of particles can be clearly observed. Moreover, aggregation occurred in all samples. These images show no change in particle morphology due to Nd loading. The shape of particles does not show much difference between the synthesis methods. However, it seems that for photocatalysts obtained by the SHT method the agglomerates are a bit smaller.

### Optical and photoluminescence properties

**Figure 3:**
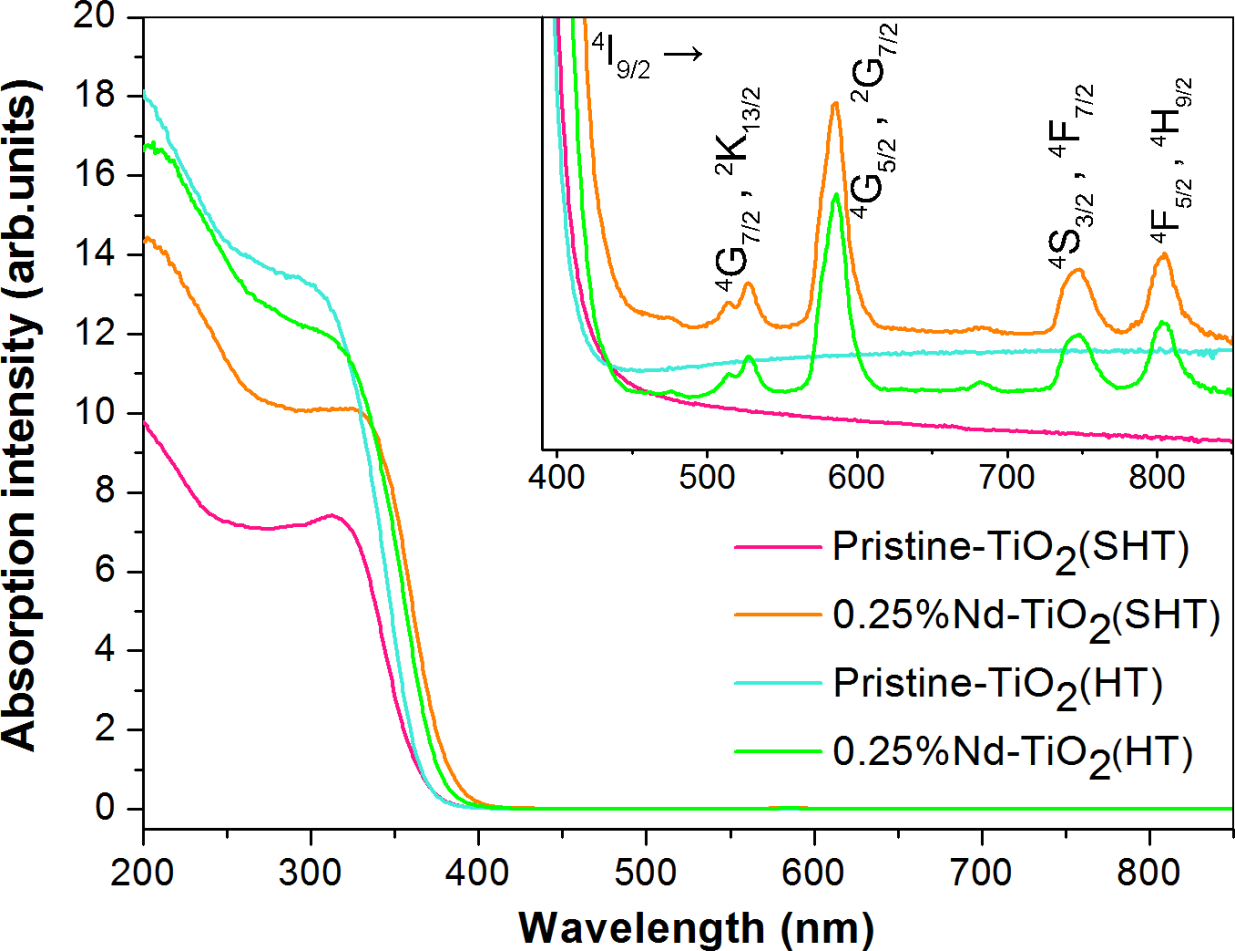
UV–vis diffuse reflectance spectra of Nd-modified TiO_2_ photocatalysts and pristine TiO_2_.

To study the optical absorption properties of the as-prepared photocatalysts, diffuse reflectance spectra (DRS) in the range of 200–850 nm were investigated, and the results are shown in Figure 3.

The optical absorption of TiO_2_ in the UV region below 400 nm can be mainly attributed to the charge transfer, related to electron excitation from the valence band to the conduction band (band-to-band transition, O_2p_→Ti_3d_) [30]. Modification of TiO_2_ with neodymium significantly affected the light absorption of the photocatalysts. A redshift of the absorption edge toward the visible region was observed for the Nd-modified samples. The optical absorption edge was shifted further when the SHT method was applied. Nassuko et al. reported that the redshift can be ascribed to the new energy level in the band gap and charge transfer between the TiO_2_ valence band and Nd^3+^ ion levels [36]. Furthermore, there are four absorption bands in the vis region typical for neodymium located at 520, 585, 745 and 805 nm. They correspond to transitions from the ^4^I_9/2_ ground state to the excited states of ^4^G_7/2_ and ^2^K_13/2_, ^4^G_5/2_ and ^2^G_7/2,_^4^S_3/2_ and ^4^F_7/2_, ^4^F_5/2_ and ^2^H_9/2_ [30,37]. In all of the studied Nd-modified samples, the intensity of these absorption bands was similar.

**Figure 4:**
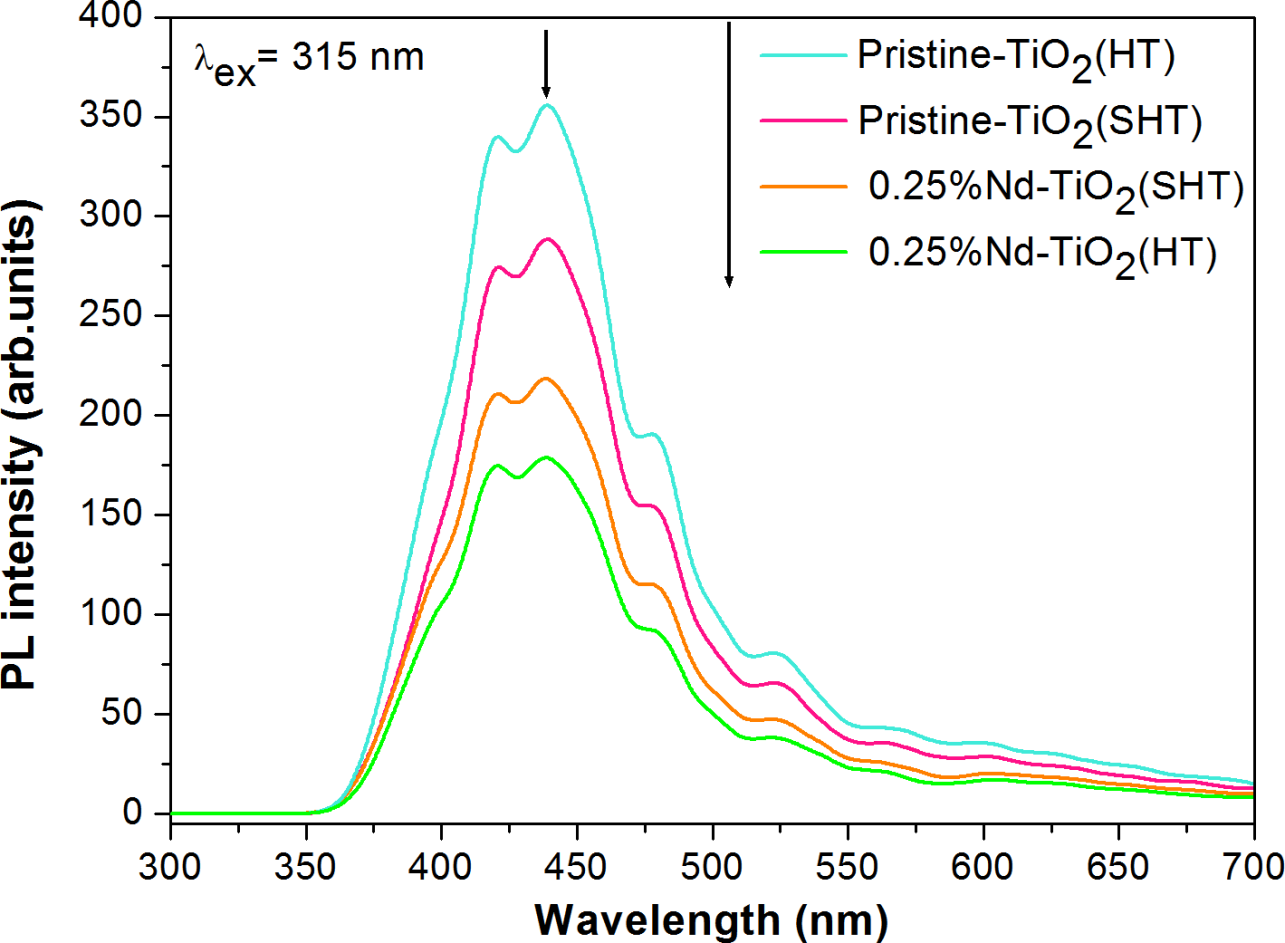
Photoluminescence spectra under UV light (λ_ex_ = 315 nm) of pristine TiO_2_ and Nd-modified TiO_2_ prepared by sol–hydrothermal and hydrothermal methods.

To understand the rate of electron–hole recombination, photoluminescence (PL) spectroscopy was applied. Figure 4 shows the PL spectra of pristine and Nd-modified TiO_2_ under excitation by light at λ_ex_ = 315 nm.

**Figure 5:**
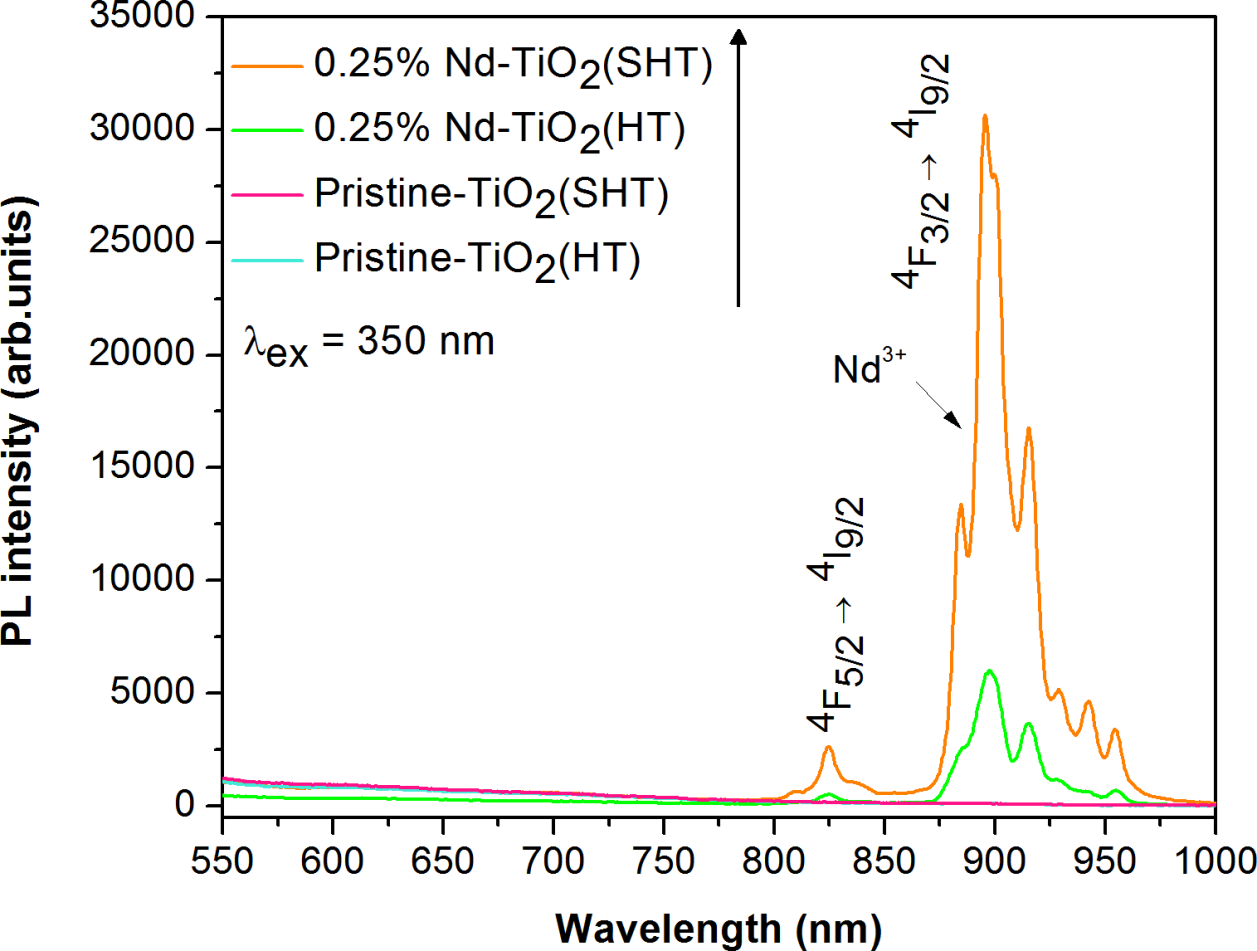
Photoluminescence spectra under laser light excitation (λ_ex_ = 350 nm) of pristine TiO_2_ and Nd-modified TiO_2_ prepared by sol–hydrothermal and hydrothermal methods.

The photoluminescence spectra show a weak broad emission band with a maximum intensity at around 350–700 nm. Khalid et al. [38] and Raja et al. [39] reported that emission in that range is characteristic for direct (band–band) or indirect (via a band-gap state) recombination of excited electrons and holes [38]. The Nd-TiO_2_ photocatalysts were characterized by a better separation ability of the photogenerated electrons and holes compared to pristine samples. In addition, the Nd-modified photocatalyst prepared by the HT method exhibited the lowest photoluminescence intensity, which suggests that the prepared material significantly inhibited the recombination of photogenerated charge carriers, which can improve the photocatalytic performance. A possible reason is the occurrence of numerous Ti–O–Nd bonds increasing the content of surface oxygen vacancies and defects [31,40]. These electron trapping sites can enhance the separation of photogenerated electron–hole pairs [24]. Furthermore, both pristine and Nd-TiO_2_ NPs exhibit obvious excitonic PL signals with a similar curve shape. The observed phenomena can be explained by the spectroscopic properties of the Nd^3+^ ions caused by the transition of electrons within the 4f subshell and the shielding properties of the 5s and 5p outer orbitals. The 4f inner shell transitions can generate different arrangements of new energy levels, improving the transfer and photogenerated lifetime of the carriers, and finally increasing the formation of highly reactive and oxidative radicals during the photocatalytic degradation [15]. The Nd-TiO_2_ NPs showed intense emission of Nd^3+^ ions under excitation by laser radiation with λ_ex_ = 350 nm (Figure 5).

The characteristic transition bands appeared in the near infrared range, with a maximum at around 900 nm (^4^F_3/2_→^4^I_9/2_ transition) and a less intense peak at 850 nm (^4^F_5/2_→^4^I_9/2_ transition) [41]. The observed emission occurred because of a direct excitation of Nd^3+^ ions into the ^4^I_9/2_→^4^D_1/2_,^4^D_3/2_,^4^D_5/2_ absorption peak [42]. The sample prepared by the HT method showed lower emission intensity than the one prepared by the SHT method. This is a result of the differences in morphology between these two samples. According to Table 1, the 0.25% Nd-TiO_2_(HT) sample exhibited crystals that are roughly half the size, which increases the number of quenching processes of Nd^3+^ on the NP surfaces. The emission of NPs is more sensitive to interactions with the surrounding environment as a larger fraction of Nd^3+^ ions can be placed on the surface in comparison to bulk material. What is more, the surface of NPs usually exhibits more defects than, which also affects the luminescence [43]. The XPS results showed a lower concentration of Nd^3+^ ions in the sample prepared by the HT method, which may be another factor responsible for its reduced emission (see below in Table 2). Furthermore, the carbon content determined by XPS is the highest for the 0.25% Nd-TiO_2_(HT) sample. Hence, organic impurities might be considered as another factor quenching the luminescence of Nd^3+^ ions. The Nd^3+^-doped samples were also irradiated with 590, 750 and 808 nm laser light, however up-conversion luminescence was not detected. The Nd^3+^ ions have dense packaged energy levels, which limits the possible efficiency of up-conversion processes [20]. To obtain up-conversion of Nd^3+^ ions, good crystallization of the host material, revealing also low phonon energy is required [20]. The presence of Nd^3+^ ions in the Nd_2_O_3_ phase or in an environment that is highly defected and contaminated by organic impurities can also hinder the up-conversion process or even make it impossible to occur.

### Chemical composition

**Figure 6:**
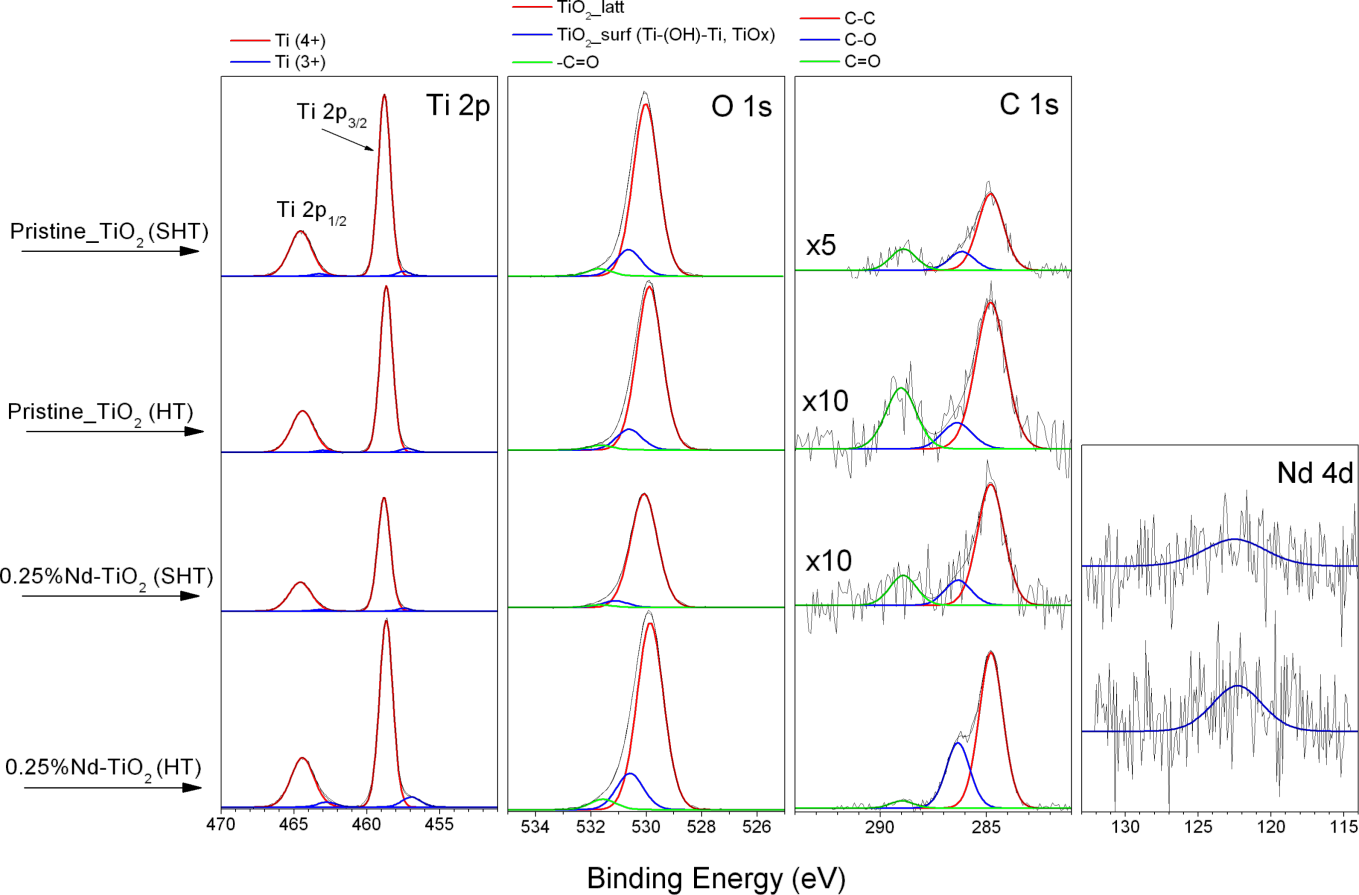
XPS spectra of pristine TiO_2_ and Nd-modified TiO_2_.

To correlate the surface properties with the photoactivity of the obtained Nd-TiO_2_, the surface layer of pristine TiO_2_ and Nd-modified TiO_2_ prepared by SHT and HT methods was examined by XPS. The existence of the titanium, oxygen and carbon has been proven in all samples and the corresponding amounts as well as the chemical character are presented in Figure 6 and in Table 2 and Table 3. The presence of neodymium was confirmed in both types of Nd-modified samples.

**Table 2:** Chemical composition of pristine and Nd-modified TiO_2_ photocatalysts based on XPS analysis.

sample	Ti (wt %)	O (wt %)	C (wt %)	Nd (wt %)	C/Ti	Nd/Ti

pristine_TiO_2_(SHT)	53.34	45.10	1.56	0	0.116	0
0.25% Nd-TiO_2_(SHT)	54.01	44.84	0.89	0.24	0.065	0.0014
pristine_TiO_2_(HT)	53.44	45.48	1.07	0	0.080	0
0.25% Nd-TiO_2_(HT)	51.19	44.11	4.54	0.17	0.353	0.0012

**Table 3:** XPS analysis data for pristine and Nd-modified TiO_2_.

sample	Ti fraction (%)		O fraction (%)			C fraction (%)		
	Ti(4+)	Ti(3+)	Ti–O_latt_	Ti–O_surf_	–C=O	C–C	C–OH	–C=O

pristine_TiO_2_(SHT)	97.11	2.89	83.50	12.88	3.62	65.50	16.07	18.43
0.25% Nd-TiO_2_(SHT)	97.48	2.52	92.59	5.40	2.01	68.80	14.25	16.95
pristine_TiO_2_(HT)	97.31	2.69	86.58	10.96	2.46	62.62	11.21	26.17
0.25% Nd-TiO_2_(HT)	92.74	7.26	79.83	15.63	4.54	68.13	28.66	3.21

Elemental surface composition and chemical character of detected elements were identified by using high-resolution (HR) XPS of the Ti 2p, O 1s, C 1s and Nd 4d orbitals. The chemical character of elements is similar to that reported in our previously published paper [23].

The neodymium content (in wt %) is consistent with the nominal amount of Nd introduced into the Nd-TiO_2_ samples during preparation via the SHT method, while it is slightly lower in the sample obtained by the HT method (Table 2). Probably, the relatively large surface content of carbon species in the last sample results in a smaller Nd concentration (compare the C/Ti and Nd/Ti atomic concentration values in Table 2). The inspection of the XPS data in Table 3 reveals that the contribution of Ti–O_surf_ (Ti–(OH)–Ti, TiO*x*) and C–OH fractions in the surface region of 0.25% Nd-TiO_2_(HT) is larger than in other samples.

This clearly indicates the higher amount of hydroxy groups on the surface of the Nd-TiO_2_ sample prepared via the HT method. The presence of the Ti–O_surf_ species can initiate the formation of Ti–O–Nd bonds. In addition, the Ti^3+^ fraction in the surface region of the 0.25% Nd-TiO_2_(HT) sample is significantly larger than in the corresponding 0.25% Nd-TiO_2_(SHT) sample (Table 3). The XPS data correspond well with the highest photocatalytic activity exhibited by the 0.25% Nd-TiO_2_ photocatalyst prepared via HT method (described in detail in the next section).

### Photocatalytic properties

**Figure 7:**
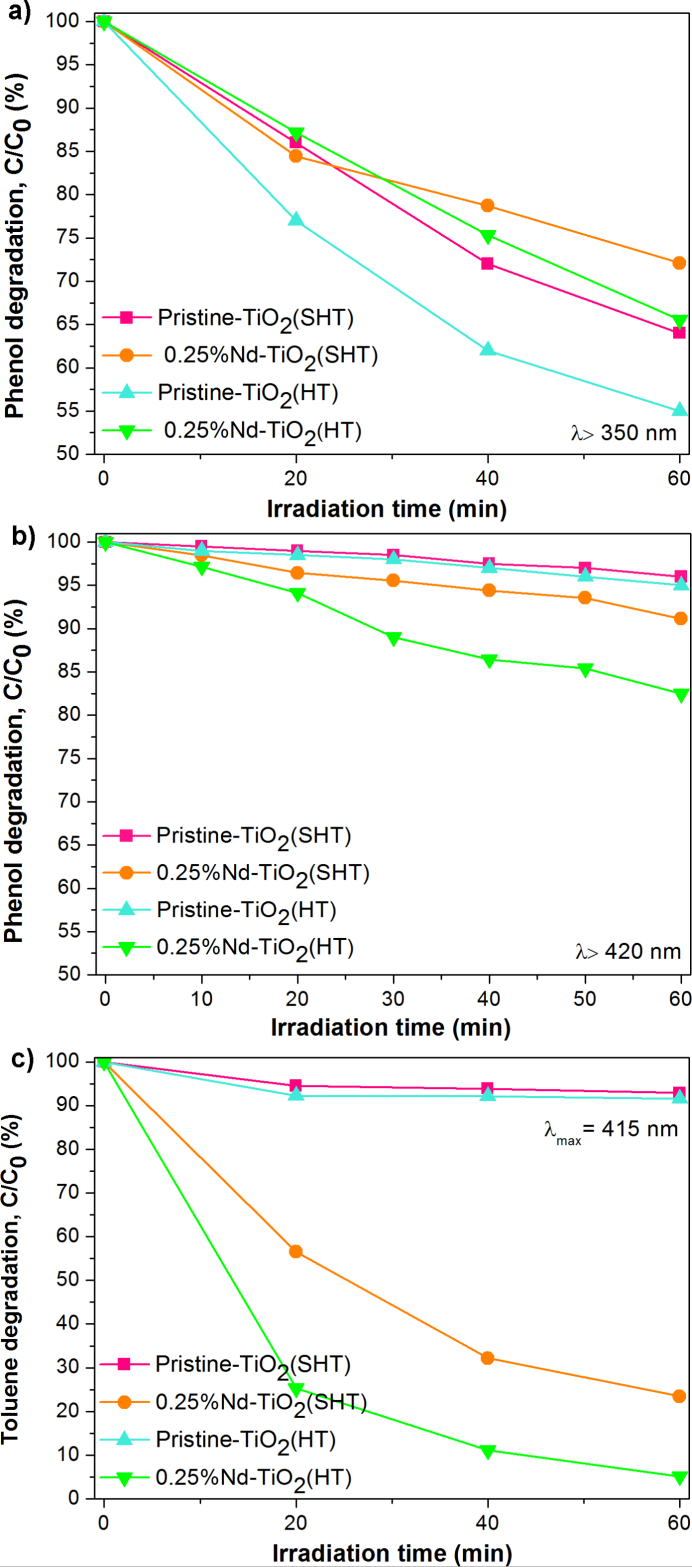
Photocatalytic activity of pristine and Nd-modified TiO_2_ NPs. Degradation of phenol in aqueous solution under (a) UV–vis and (b) vis irradiation, and degradation of gaseous toluene under (c) vis irradiation.

The photocatalytic properties of pristine and Nd-modified TiO_2_ were investigated using the photocatalytic degradations of phenol in aqueous solution and of gaseous toluene. Kinetics and degradation rate of model pollutants are presented in Figure 7 and Table 4.

**Table 4:** Photocatalytic activity under UV–vis and vis irradiation of pristine and Nd-modified TiO_2_ photocatalysts.

sample label	toluene degradation rate (μmol·dm^−1^·min^−1^) under vis (λ_max_ = 415 nm) irradiation	phenol degradation rate (μmol·dm^−1^·min^−1^)		phenol degradation rate (μmol·dm^−1^·min^−1^) with added scavenger under vis (λ > 420 nm) irradiation			
		under UV–vis (λ > 350 nm) irradiation	under vis (λ > 420 nm) irradiation	silver nitrate	ammonium oxalate	benzoquinone	*tert*-butanol

pristine-TiO_2_(SHT)	0.25	1.28	0.14	—	—	—	—
0.25% Nd-TiO_2_(SHT)	2.71	0.99	0.31	0.12	0.30	0.22	0.30
pristine-TiO_2_(HT)	0.30	1.59	0.18	—	—	—	—
0.25% Nd-TiO_2_(HT)	3.36	1.22	0.62	0.15	0.58	0.21	0.57

The photodegradation of phenol was performed in aqueous solution under UV–vis (λ > 350 nm) and vis (λ > 420 nm) light irradiation. The data showed that the photocatalytic activitiy of Nd-modified TiO_2_ NPs were lower than that of pristine TiO_2_ under UV–vis irradiation. The highest activity was observed for pristine TiO_2_ prepared via the HT method, with the phenol degradation rate reaching 1.59 μmol·dm^−1^·min^−1^. In the case of Nd-modified samples, the photocatalysts prepared via the HT method also showed higher activity (the degradation rate was equal to 1.22 μmol·dm^−1^·min^−1^). It should be pointed out that samples prepared by the HT process revealed the highest inhibition of recombination of the photogenerated charge carriers (see Figure 4). The observed decrease of photocatalytic activity of Nd-modified TiO_2_ is due to the presence of Nd^3+^ ions on the surface of the TiO_2_ NPs, which lowered the excitation under UV irradiation. The UV irradiation does not yield sufficiently high energy of photons to penetrate deeper into the surface of the material [11]. This phenomenon can also be explained by the spectroscopic properties of rare earth ions. In our previous paper [23], it has been proven that Nd^3+^ ions are prone to cross-relaxation (energy migration between Nd^3+^ ions). Therefore, Nd^3+^ ions could absorb UV irradiation and emit energy in radiative transitions (luminescence). The photocatalytic properties of the as-prepared samples in the aqueous phase were also investigated under visible light irradiation (Figure 6b). The Nd-modified TiO_2_ has the ability to degrade effectively phenol under visible light, while pristine TiO_2_ shows only weak activity. The photocatalyst 0.25%Nd-TiO_2_(HT) exhibited the highest photocatalytic activity with a phenol degradation rate of 0.62 μmol·dm^−1^·min^−1^. Moreover, the lower photocatalytic activity of Nd-TiO_2_ sample prepared via the SHT method under UV–vis and vis irradiation (0.99 and 0.31 μmol·dm^−1^·min^−1^, respectively), is the consequence of a small amount of surface adsorbed hydroxy groups. A similar decrease of photoactivity for RE-modified TiO_2_ was reported in our previous paper [24]. It was shown that photocatalysts prepared by the sol–gel method contain less OH groups than the powders obtained via the hydrothermal method, and the latter exhibited lower activity under UV–vis light irradiation. This behaviour was also observed by Tobaldi and co-workers [44]. They concluded that the overall photocatalytic activity is strongly influenced by surface hydroxyl groups helping to generate reactive oxygen species, such as hydroxyl radicals.

**Figure 8:**
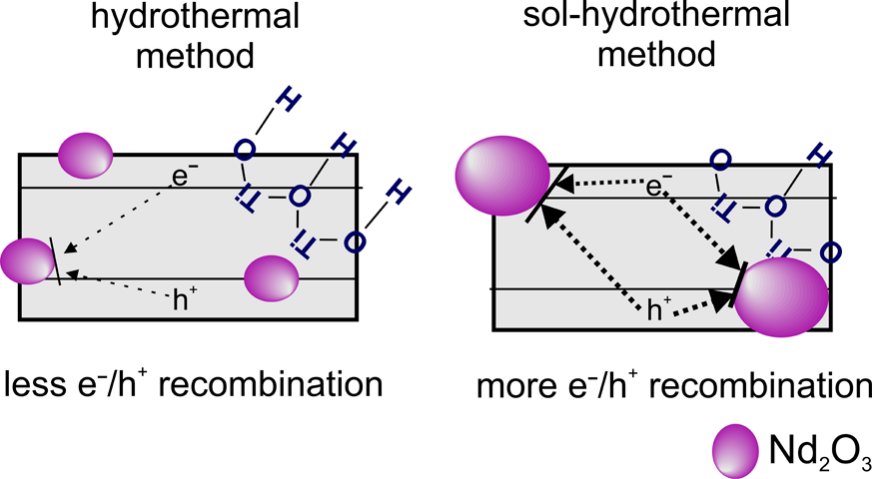
Schematic illustration showing the impact of preparation methods on the surface properties and the charge carrier recombination processes.

The photocatalytic degradation of toluene was carried out under LED irradiation (λ_max_ = 415 nm). A high photocatalytic activity was exhibited by all the neodymium-modified photocatalysts (Figure 6c). The rate of toluene degradation was 3.36 μmol·dm^−1^·min^−1^ for HT-prepared TiO_2_ modified with Nd, while the photodegradation efficiency decreased in SHT-prepared TiO_2_ down to 2.71 μmol·dm^−1^·min^−1^. Pristine TiO_2_ exhibits only low activity (Figure 6c). As photolysis of toluene was not observed and the toluene concentration decreased in the dark experiment by approximately 6.5% for all samples, probably pollutant was adsorbed on the surface of TiO_2_ and on the walls of the reactor. A correlation between photocatalytic activity measured in both model reactions and the BET surface area was not observed. Based on XPS analysis, it can be seen that photocatalysts prepared via the HT method had a higher amount of hydroxy groups on the surface than those obtained by the SHT method. The hydroxy groups can act as adsorption centres on which the degradation of pollutants takes place [45]. Moreover, a high amount of oxygen vacancies and defects was found on the surface of the 0.25% Nd-TiO_2_(HT) sample. As previously mentioned, the presence of oxygen deficient centres on the surface and surface defects such as Ti^3+^ can reduce the rate of electron–hole pair recombination [45–47]. Further, the light adsorption ability could be strongly related to the band-gap structure, which is affected by defects. With a reduced band gap or addition of sub-bands, TiO_2_ can respond to visible light, increasing its light absorption efficiency [48]. A schematic illustration showing impact of the preparation methods on the surface properties and the charge carrier recombination processes is presented in Figure 8.

The results of the photodegradation significant differences between aqueous solution and gas phase. The quantum yield of the reaction in the gas phase is much higher than that one in aqueous solution due to lower light scattering. Also, a lower irradiation intensity is required to perform the photocatalytic reaction in the gas phase [8,49].

### Degradation mechanism

**Figure 9:**
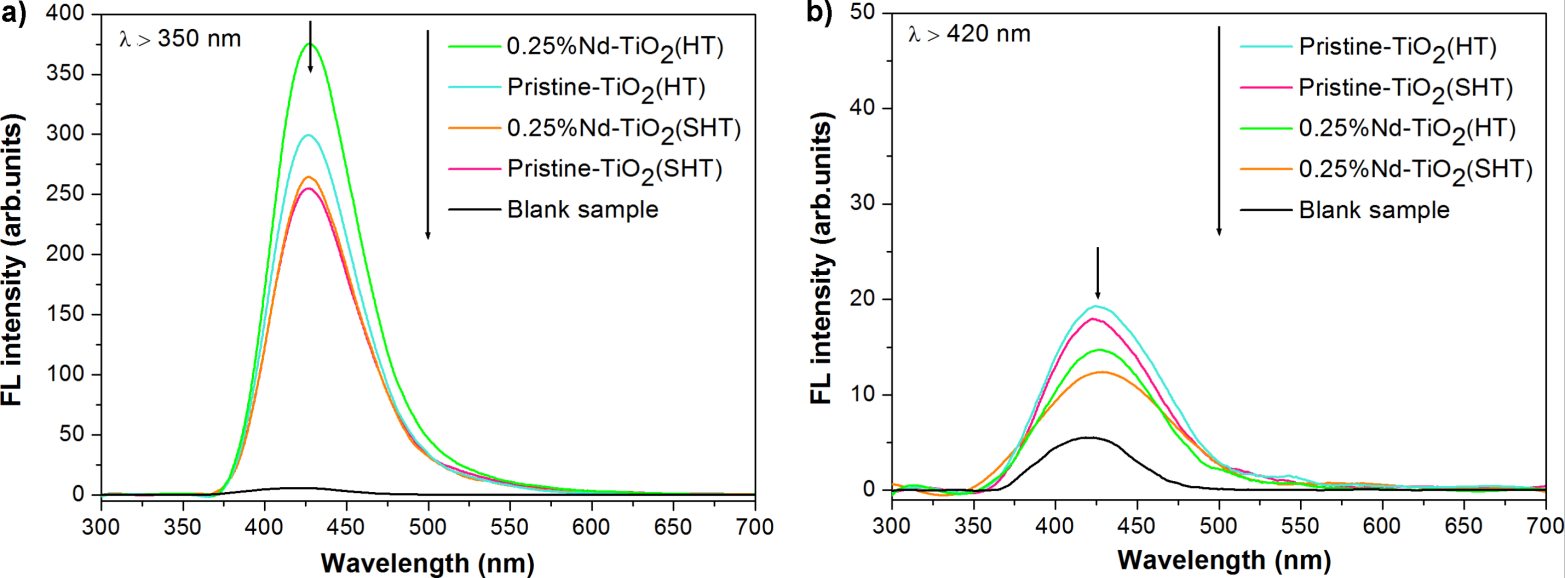
Fluorescence spectral changes in a solution of terephthalic acid under (a) UV–vis (λ > 350 nm) and (b) vis (λ > 420 nm) light irradiation.

Based on the obtained results, it can be stated that the Nd-modified TiO_2_ could be effectively excited by irradiation with visible light. To explore which active species are responsible for the degradation of phenol under vis irradiation, hydroxyl radical generation tests with terephthalic acid (TPA) were performed. Figure 9 shows the fluorescence of 2-hydroxyterephthalic acid after 60 min in the presence of pristine and Nd-modified TiO_2_ under UV–vis and vis light. The increase in intensity of the fluorescent peak indicates the formation of ^•^OH radicals produced in the aqueous solution.

As shown in Figure 9a, the presence of Nd^3+^ increases the intensity of the peaks. This indicates that Nd^3+^ effectively leads to an increase in the production of hydroxyl radicals on the surface of the photocatalysts under UV–vis light irradiation. Moreover, the obtained results showed that the intensity of the FL spectra increases for the HT series of photocatalysts. The obtained results correlate with photocatalytic activity under UV–vis light. It should be mentioned that the number of hydroxyl radicals is increased under the influence of UV–vis irradiation in the presence of Nd-modified TiO_2_ compared to pristine TiO_2_. However, the observed efficiency of phenol degradation in presence of Nd-TiO_2_ was much lower. The difference is caused by the different reaction paths of phenol photodegradation and formation of 2-hydroxyterephthalic acid from TPA through generated ^•^OH radicals. Based on the available literature, the TPA formation depends only on the amount of ^•^OH radicals generated at the surface of the excited semiconductor. The phenol degradation can result from: (i) the reaction of ^•^OH with phenol, (ii) the reaction of phenol with photogenerated holes in TiO_2_, and (iii) by direct oxidation of phenol through oxygen dissolved in water [50]. When the TPA test was carried out under vis irradiation (Figure 9b), the FL intensity was close to zero for all samples. Based on the presented results it can be stated that the modification of TiO_2_ with Nd ions does not increase the production of hydroxyl radicals under visible light irradiation. According to our previous paper, the photodegradation of phenol under vis light irradiation occurs through the other forms of reactive oxygen species such as O_2_^•−^, HO_2_^•^ and H_2_O_2_ [8,23].

**Figure 10:**
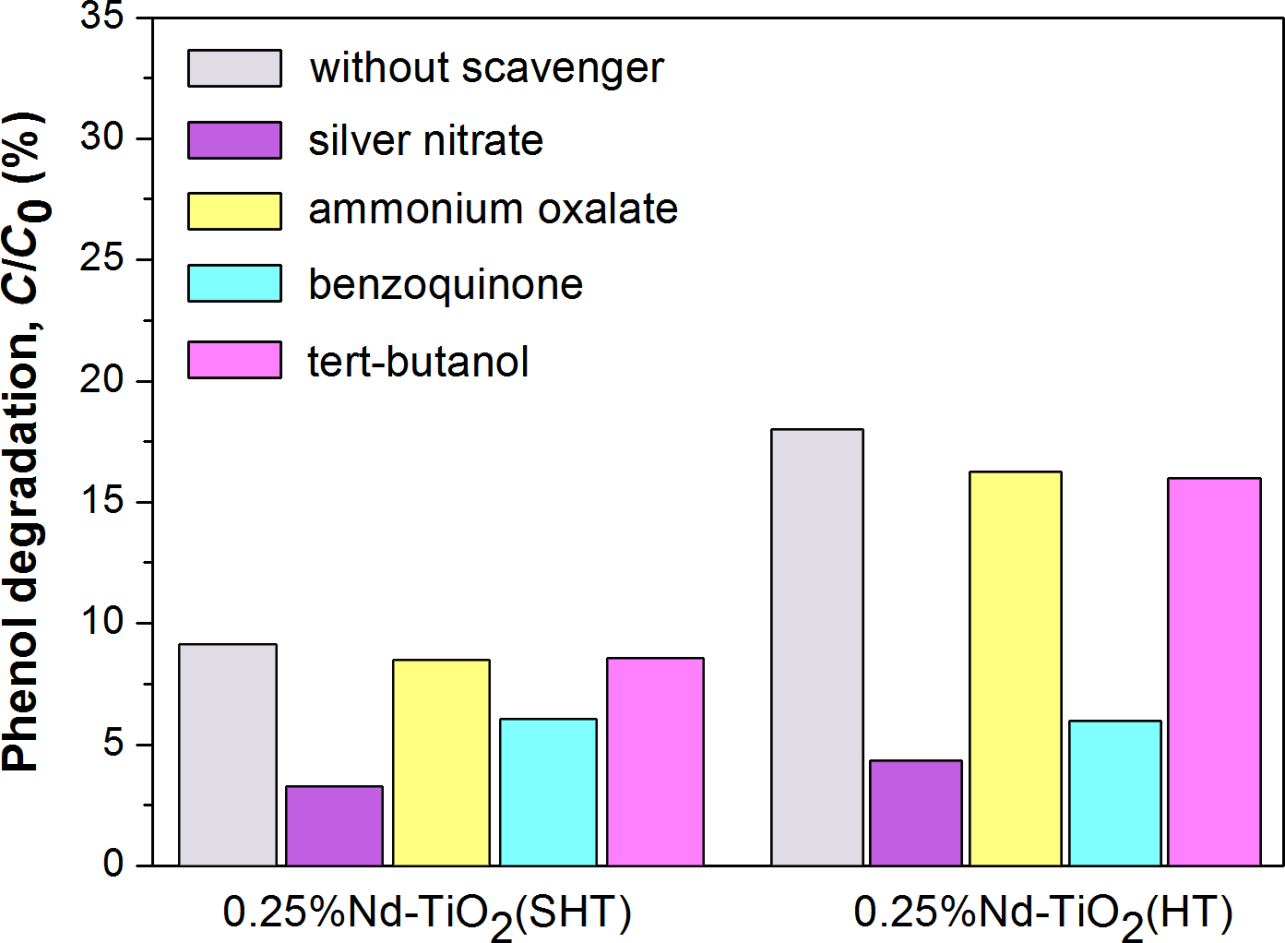
Photocatalytic decomposition of phenol in the presence of Nd-modified TiO_2_ and scavengers after 60 min of visible (λ > 420 nm) irradiation.

In order to better understand which reactive oxygen species may be important in the photocatalytic process, the photocatalytic activity tests in aqueous solution have been carried out in the presence of Nd-TiO_2_ and different scavengers (Figure 10).

Silver nitrate was used as electron scavenger, ammonium oxalate as hole scavenger, benzoquinone for O_2_^•−^ and *tert*-butanol for ^•^OH radicals. After 60 min of visible light irradiation in the presence of SHT photocatalyst, the degradation rate declined from 0.31 to 0.30 μmol·dm^−1^·min^−1^ due to addition of ammonium oxalate and *tert*-butanol (Table 4). While, after the addition of silver nitrate and benzoquinone, the degradation rate decreased from 0.31 to 0.12 and 0.22 μmol·dm^−1^·min^−1^, respectively, suggesting that photogenerated electrons and superoxide radicals are the main active species responsible for the degradation of the model pollutants. In the presence of HT photocatalyst with scavengers similar phenomena were observed. The photocatalytic system with added ammonium oxalate and *tert*-butanol showed congruous degradation rates (0.58 and 0.57 μmol·dm^−1^·min^−1^, respectively) compared to the system without scavengers (0.62 μmol·dm^−1^·min^−1^), suggesting a limited role played by holes in the photocatalytic process. The addition of silver nitrate and benzoquinone significantly reduced the phenol degradation rate (from 0.62 to 0.15 and 0.21 μmol·dm^−1^·min^−1^, respectively), indicating that superoxide radical is the major active species in this system. These experiments also showed that for the photodegradation of phenol with the photocatalyst prepared via HT method, a larger suppression was noted upon the addition of benzoquinone, compared to the photocatalyst prepared via SHT method. This suggests that 0.25% Nd-TiO2(HT) generates a bigger amount of O_2_^•−^. The reaction of electrons in the conduction band of TiO_2_ is probably responsible for the formation of O_2_^•−^. Electrons from the conduction band of TiO_2_, generated by the excitation of the Nd^3+^, migrate to the surface of the semiconductor where they are involved in the formation of O_2_^•−^ and then H_2_O_2_ and HO_2_^•^, as presented in the following reactions [8]:


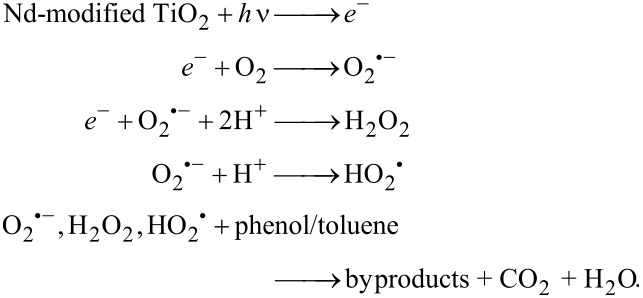


**Figure 11:**
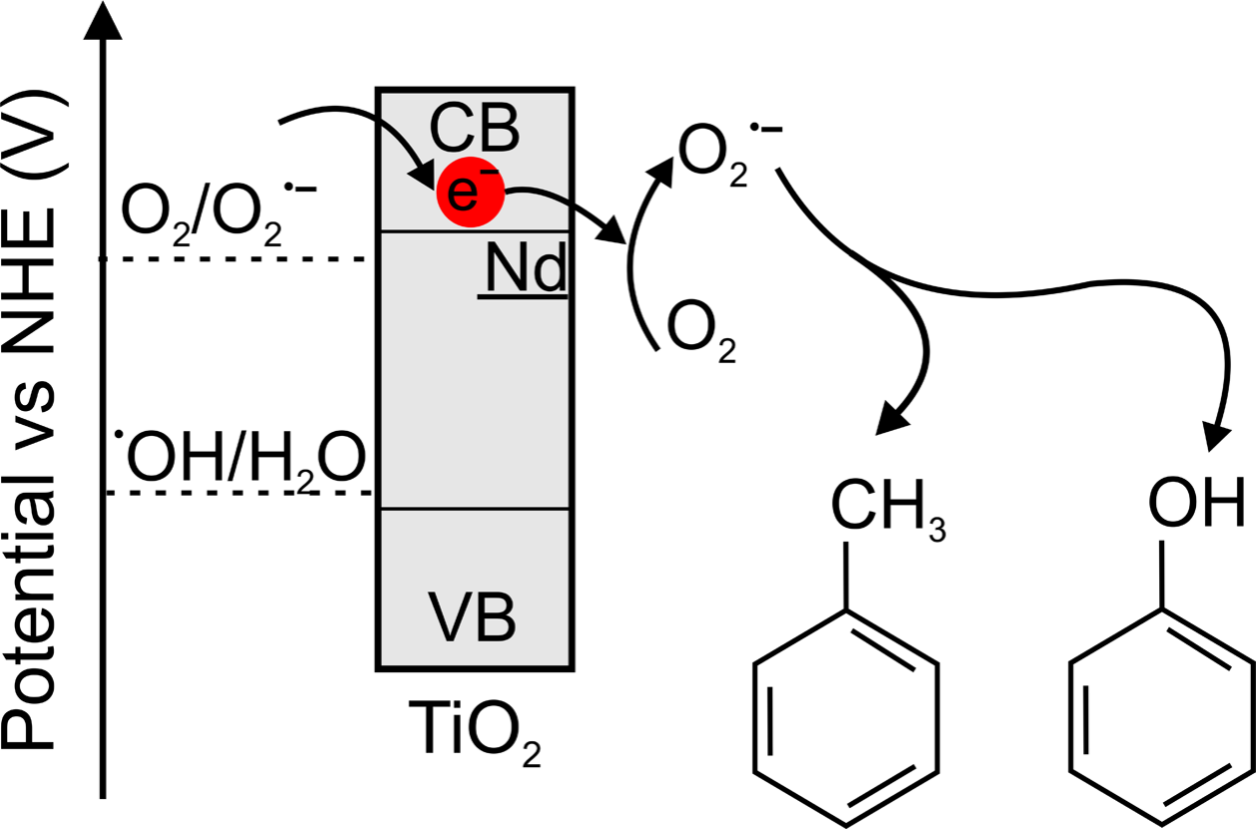
Proposed photocatalytic mechanism of Nd-modified TiO_2_ under visible light.

The proposed photocatalytic mechanism under visible light can be explained by the energy diagram shown in Figure 11.

## Conclusion

We have observed how the spectroscopic properties of Nd^3+^ ions on the surface of TiO_2_ NPs can be conveniently exploited for a variety of environmental purification processes. We presented the experimental studies concerning the effect of synthesis methods on the photocatalytic activity of Nd-modified TiO_2_. Nanocrystalline anatase-phase TiO_2_ NPs were successfully prepared via hydrothermal and sol–hydrothermal methods. Both Nd-modified samples can achieve the photocatalytic degradation of phenol and toluene under visible light, and Nd-TiO_2_ has a higher activity than pristine TiO_2_ (2.25- and 6.3-times higher activity for 0.25% Nd-TiO_2_(SHT), and 3.6- and 7.1-times higher activity for 0.25% Nd-TiO_2_(HT), respectively). This is mainly due to the increase of the BET surface area, the decrease of the anatase crystallite size and the prevention of recombination of charge carriers. The surface properties of the Nd-TiO_2_ NPs obtained via hydrothermal method yield a significantly higher photocatalytic activity compared to Nd-TiO_2_ NPs prepared via sol–hydrothermal process. The photocatalyst prepared via HT method has three times more hydroxy groups on its surface layer and a two times higher amount of surface defects than the powder obtained by SHT method. According to the photocatalytic degradation experiments, the Nd-modified TiO_2_ photocatalyst preparated via HT method can be effectively used for air purification using LED irradiation (the degradation rate of toluene reached 3.36 μmol·dm^−1^·min^−1^). Furthermore, photocatalytic activity test in the presence of Nd-TiO_2_ and scavengers confirm that superoxide radicals were responsible for the visible-light degradation of the model pollutant in aqueous solution.

## Experimental

### Materials

Titanium(IV) butoxide (97%, TBOT) and Nd(NO_3_)_3_·6H_2_O (99.99%) were purchased from Sigma-Aldrich, Poland. Nitric acid (65%) and hydrochloric acid (31–38%) were purchased from STANLAB, Poland. Ethanol, phenol and terephthalic acid were purchased from POCh S.A, Poland. P25 from Evonik, Germany was used as a standard and for comparison of the photocatalytic activity. Deionised water (0.05 µS) was used for all reactions and treatment processes. All the chemicals were used as received without further purification.

### Preparation of Nd-TiO_2_

Nd-TiO_2_ NPs were synthesized by two methods, i.e., hydrothermal (HT) and sol–hydrothermal (SHT).

### HT method

In a typical procedure, 15 mL of TBOT was dissolved in 80 mL of ethanol. Then, 40 mL water (pH 3 adjusted with HCl) was added dropwise. Next, a certain amount of solid Nd(NO_3_)_3_·6H_2_O was added. The mixture was stirred for 30 min at room temperature and treated at 180 °C for 6 h in a 200 mL autoclave.

### SHT method

In the first step, 24 mL TBOT was dissolved in 48 mL ethanol (solution A), and stirred for 15 min. Then, to 48 mL ethanol, 20 mL water, and 1.90 mL HNO_3_ was added a certain amount of solid Nd(NO_3_)_3_·6H_2_O (solution B). In the next step, solution B was added to solution A dropwise and stirred for 30 min at room temperature. The obtained sol was treated at 160 °C for 3 h in a 200 mL autoclave.

The samples obtained from both methods were separated by centrifugation, washed three times with deionised water and ethanol, and dried at 35 °C for 16 h and ground. Crystallization of titania was performed at a temperature of 450 °C for 2 h in a muffle furnace under air atmosphere with a heating rate of 2 °C/min followed by grinding to obtain the Nd-TiO_2_ powders. For comparison, TiO_2_ without Nd was prepared likewise. A description of the prepared photocatalysts is shown in Table 1.

### Characterization of Nd-TiO_2_

X-ray diffraction (XRD) was used to determine and verify the crystalline structure of the obtained photocatalysts. XRD patterns were recorded on a Rigaku diffractometer (RINT Ultima+) equipped with a graphite monochromator using Cu Kα radiation (40 kV tube voltage and 20 mA tube current). Measurements were performed in a 2θ range of 5–90°. Based on the results obtained, the particle sizes of photocatalysts and XRD data were calculated using the Scherrer equation.

Nitrogen adsorption–desorption isotherms were measured at −196 °C (liquid nitrogen temperature) using a Micromeritics Gemini V (model 2365). The surface area was determined according to the standard Brunauer–Emmet–Teller (BET) method. All samples were degassed at 200 °C prior to nitrogen adsorption measurements.

The morphology of obtained Nd-TiO_2_ was determined using scanning electron microscopy (SEM, FEI Quanta 250 FEG).

UV–vis diffuse reflectance spectra of the synthesized materials were recorded in the range of 200–850 nm with a scanning speed of 200 nm/min at room temperature using a Shimadzu UV–vis spectrophotometer (UV 2600) equipped with an integrating sphere. BaSO_4_ was used as the reference.

Photoluminescence (PL) properties in the range of 300–700 nm were measured on a Perkin Elmer limited LS50B spectrophotometer equipped with a xenon discharge lamp as an excitation source and an R928 photomultiplier as a detector. The spectra were recorded with an excitation wavelength of 315 nm directed on the sample surface at an angle of 90°. PL spectra in the range covering Nd^3+^ emission (550–1000 nm) were measured with a Princeton Instrument PIXIS:256E digital CCD camera, equipped with a SP-2156 Imaging Spectrograph and an Opolette 355LD UVDM tunable laser as the excitation source.

X-ray photoelectron spectroscopy (XPS) measurements were used for chemical characterization of the Nd-TiO_2_ composites surface. The high-resolution (HR) XPS spectra were recorded on PHI 5000 VersaProbe (ULVAC-PHI) spectrometer with monochromatic Al Kα radiation (*h*ν = 1486.6 eV). The binding energy (BE) scales of all detected HR spectra were calibrated by centering the aliphatic carbon peak (C–C) at 284.8 eV.

### Measurement of photocatalytic activity

The photocatalytic activity of the obtained Nd-TiO_2_ samples was studied in two model processes, namely decomposition of phenol in aqueous solution and degradation of gaseous toluene. In addition, to determine which reactive oxygen species participate in the degradation mechanism, a hydroxyl radical test using terephthalic acid and a reactive oxygen species formation test using benzoquinone, silver nitrate, ammonium oxalate and *tert*-butanol as scavengers were carried out.

Measurements of phenol decomposition and generation of ^•^OH radicals were carried out for two irradiation ranges, UV–vis (λ > 350 nm) and vis (λ > 420 nm), while scavenger test was carried under vis (λ > 420 nm) light irradiation, using a 1000 W Xenon lamp (Oriel 66021). The optical path included a water filter to cut off IR irradiation. For the test of UV–vis and vis light-induced activity the light beam passed through GG350 or GG420 filters to cut-off wavelengths shorter than 350 or 420 nm, respectively. The experimental procedure was as follows: Nd-TiO_2_ powder (125 mg) was suspended in aqueous solutions of phenol (0.21 mM) or terephthalic acid (0.5 mM) or in an aqueous solution of scavenger and phenol in a quartz photoreactor (working volume of about 25 mL). After 30 min of mixing (550 rpm) and aeration (5 dm^3^/h) in the dark, the suspension was irradiated with a cut-off spectrum of light. The temperature of the suspension during photoirradiation was maintained at 10 °C using a thermostatically controlled water bath (to avoid the stripping process). Samples of 0.5 mL reaction mixture were collected after regular time periods during irradiation and filtered through syringe filters (diameter 0.2 μm) to remove the suspended photocatalyst particles. Kinetics of phenol degradation were determined by high-performance liquid chromatography (HPLC, Shimadzu). The HPLC system was equipped with a Kinetex C18 column (150 mm × 3 mm; particle size of 2.6 μm; pore diameter 100 Å) and the SPD-M20A diode array detector (λ = 205 nm). The flow rate was maintained at 0.4 mL/min with a mobile phase composed of acetonitrile and water (7.5/92.5 v/v). The injection volume was 30 μL. For fluorescnece measurements, 2 mL of the reaction mixture of terephthalic acid were collected after 60 min irradiation and filtered through syringe filters (diameter 0.2 μm) to remove the suspended photocatalyst particles. Fluorescence spectra were measured at room temperature using a Perkin Elmer limited LS50B spectrophotometer equipped with a xenon discharge lamp as an excitation source and an R928 photomultiplier as a detector. The obtained solution was measured with an excitation wavelength of 315 nm.

Decomposition of toluene in the gas phase was carried out in a flat stainless steel reactor with the working volume of about 35 mL equipped with a quartz window, two valves and a septum. An array of five LEDs (λ_max_ = 415 nm) was used as an irradiation source. A glass plate covered by Nd-modified TiO_2_ was placed at the bottom side of the reactor followed by closing the reactor with a quartz window. Subsequently, the gaseous mixture was passed through the reaction chamber for 1 min. After closing the valves, the reactor was kept in dark for 30 min in order to achieve adsorption equilibrium. A reference of 200 μL sample was taken just before starting irradiation and further samples were collected after regular time periods during irradiation. The analysis of toluene concentration in the gas phase was performed using a gas chromatograph (Trace 1300, Thermo Scientific) equipped with a flame ionization detector (FID) and Elite-5 capillary column.
